# Computed tomography guided electromagnetic navigation system in percutaneous laser ablation for treating primary lung cancer: a case report

**DOI:** 10.3389/fonc.2024.1396452

**Published:** 2024-05-31

**Authors:** Xiaodan Liu, Shusen Zhang, Honglin Li, Xuezhu Ren, Xiaolan Xu, Xuejing Wang, Liyun Ye, Zhigang Cai

**Affiliations:** ^1^ The First Department of Pulmonary and Critical Care Medicine, The Second Hospital of Hebei Medical University, Shijiazhuang, China; ^2^ Department of Pulmonary and Critical Care Medicine, Affiliated Xing Tai People Hospital of Hebei Medical University, Xingtai, China; ^3^ Postdoctoral Mobile Station, Hebei Medical University, Shijiazhuang, China; ^4^ Hebei Province Xingtai People’s Hospital Postdoctoral Workstation, Xingtai, China

**Keywords:** computed tomography, electromagnetic navigation system, percutaneous, laser ablation, primary lung cancer

## Abstract

**Background:**

The majority of patients of lung cancer have already lost the chance of surgery at the time of diagnosis. Percutaneous local thermal ablation is a precise minimally invasive technique and a viable alternative to surgical treatment. Compared with radiofrequency ablation and microwave ablation, percutaneous laser ablation for the treatment of lung tumors is less commonly used and reported, especially for primary lung cancer.

**Case presentation:**

A 63-year-old male patient with mixed pulmonary nodules selected computed tomography-guided electromagnetic navigation system for percutaneous biopsy and laser ablation therapy. The puncture point was determined through Computed tomography scanning, along with the placement of the electromagnetic navigation system locators. After rapid on-site evaluation and pathological examination of the puncture tissue specimen, the diagnosis of lung adenocarcinoma was confirmed. A 980-nanometer wavelength semiconductor laser fiber was inserted into the appropriate position guided by the electromagnetic navigation system. Subsequently, a power of 7 watt was applied to ablate the tumor for 30 seconds, then pause for 60 seconds before repeating the procedure. Positron emission tomography-Computed tomography examination was performed 1 month after operation, suggesting complete response of the tumor.

**Conclusion:**

Here, we present a case of percutaneous laser ablation treatment for primary lung cancer guided by computed tomography-electromagnetic navigation system. As a more precise, shorter duration, impedance-independent, safe and effective minimally invasive thermal ablation method, it is expected to gain wider application and become a novel alternative for surgical treatment.

## Introduction

Surgical resection is widely recognized as the preferred treatment modality for lung cancer, particularly in cases of early-stage lung cancer (1). However, approximately 80% of patients have already lost the opportunity for surgical treatment at the time of diagnosis. Even in stage I non-small cell lung cancer, up to one-fourth of patients are unable to undergo surgical treatment, possibly due to advanced age, solitary lung, poor cardiopulmonary function, comorbidities, or refusal of surgery (2, 3).

Percutaneous local thermal ablation is a precise minimally invasive technique that has gained increasing attention as an alternative to surgical treatment (4). Thermal ablation mainly includes radiofrequency ablation (RFA), microwave ablation (MWA), laser ablation (LA), and high-intensity focused ultrasound (HIFU), which utilize probes to reach the tumor and directly kill tumor cells within one or more lesions through the biological thermal effect. Due to the air-insulating property of the lung, more heat can be focused on the tumor tissue during local thermal ablation. Moreover, normal lung tissue has a faster healing rate after thermal injury. Therefore, compared to other combinations or organs, local thermal ablation of the lung can achieve a larger ablation volume and less damage to surrounding normal tissues (5). Compared to RFA and MWA, percutaneous laser ablation (PLA) therapy for lung tumors started relatively late and has been the subject of relatively few studies. However, PLA offers advantages such as more precise ablation zone, shorter ablation time, no impedance effect, and real-time monitoring under magnetic resonance imaging (MRI) guidance, which has gradually attracted the attention of clinical practitioners (4, 6, 7).

The laser is a monochromatic light emitted at a specific wavelength and can rapidly heat the tissue to above 60°C through continuous or pulsed release, causing coagulative necrosis and thus destruction of tumor tissue (8, 9). Early research reports have preliminarily confirmed the safety and efficacy of PLA therapy for pulmonary metastases under image guidance (10–12). However, percutaneous laser ablation of primary lung tumors has rarely been reported. This case reports the diagnosis and treatment process of a patient with lung adenocarcinoma who underwent PLA guided by the computed tomography (CT)-electromagnetic navigation system (ENS) at the Second Hospital of Hebei Medical University.

## Case presentation

**Figure 1:**
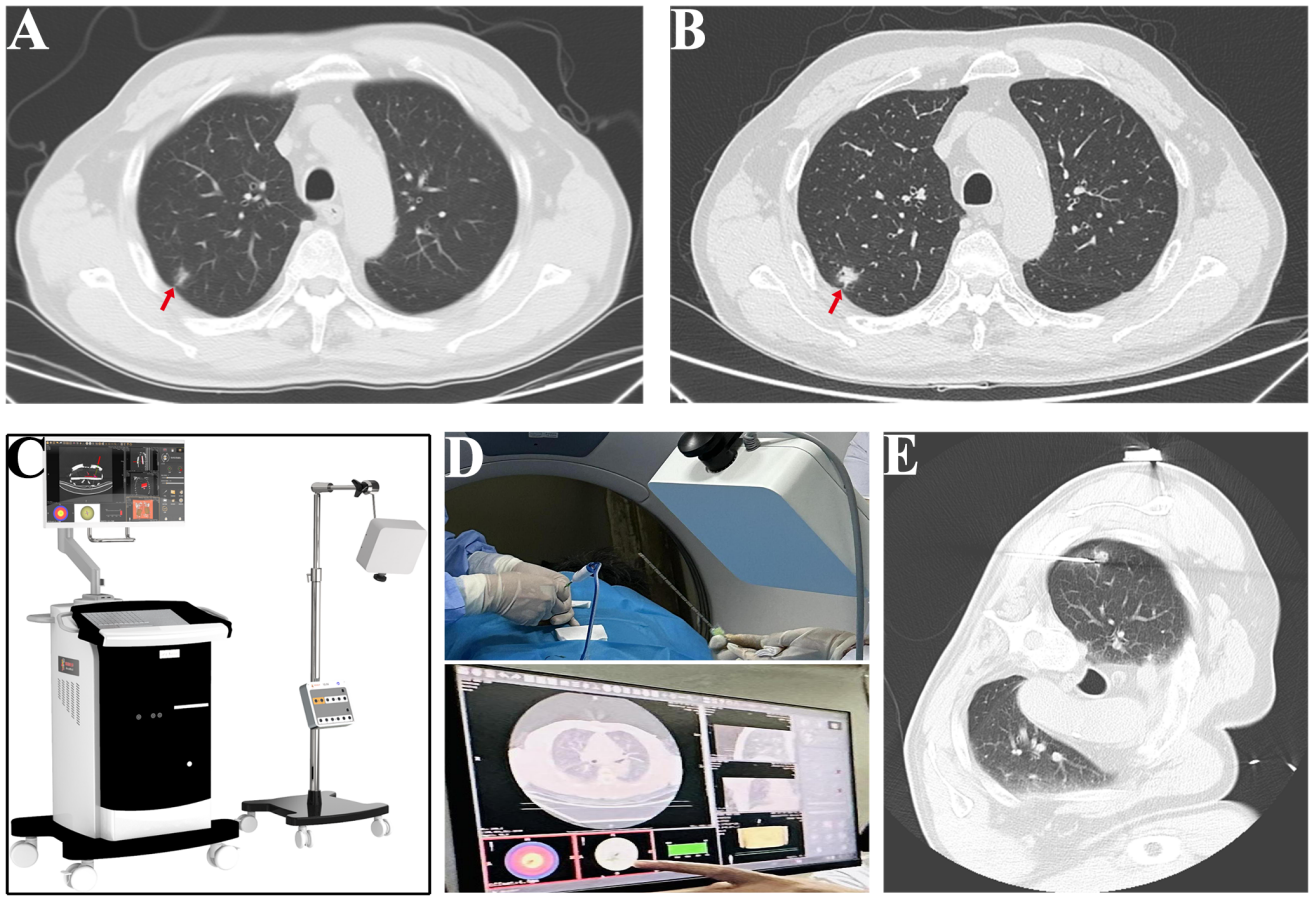
Process of CT-electromagnetic navigation system guided percutaneous laser ablation. **(A)** The size of the pulmonary nodule was measured at 8.5mm×6.1mm in March (red arrow). **(B)** The size of the pulmonary nodule was measured at 18mm×19mm in September (red arrow). **(C)** Image of electromagnetic navigation system equipment. **(D)** Representative images of the puncture process guided by CT electromagnetic navigation system. **(E)** Image of successful positioning of the puncture sheath to the tumor site.

**Figure 2:**
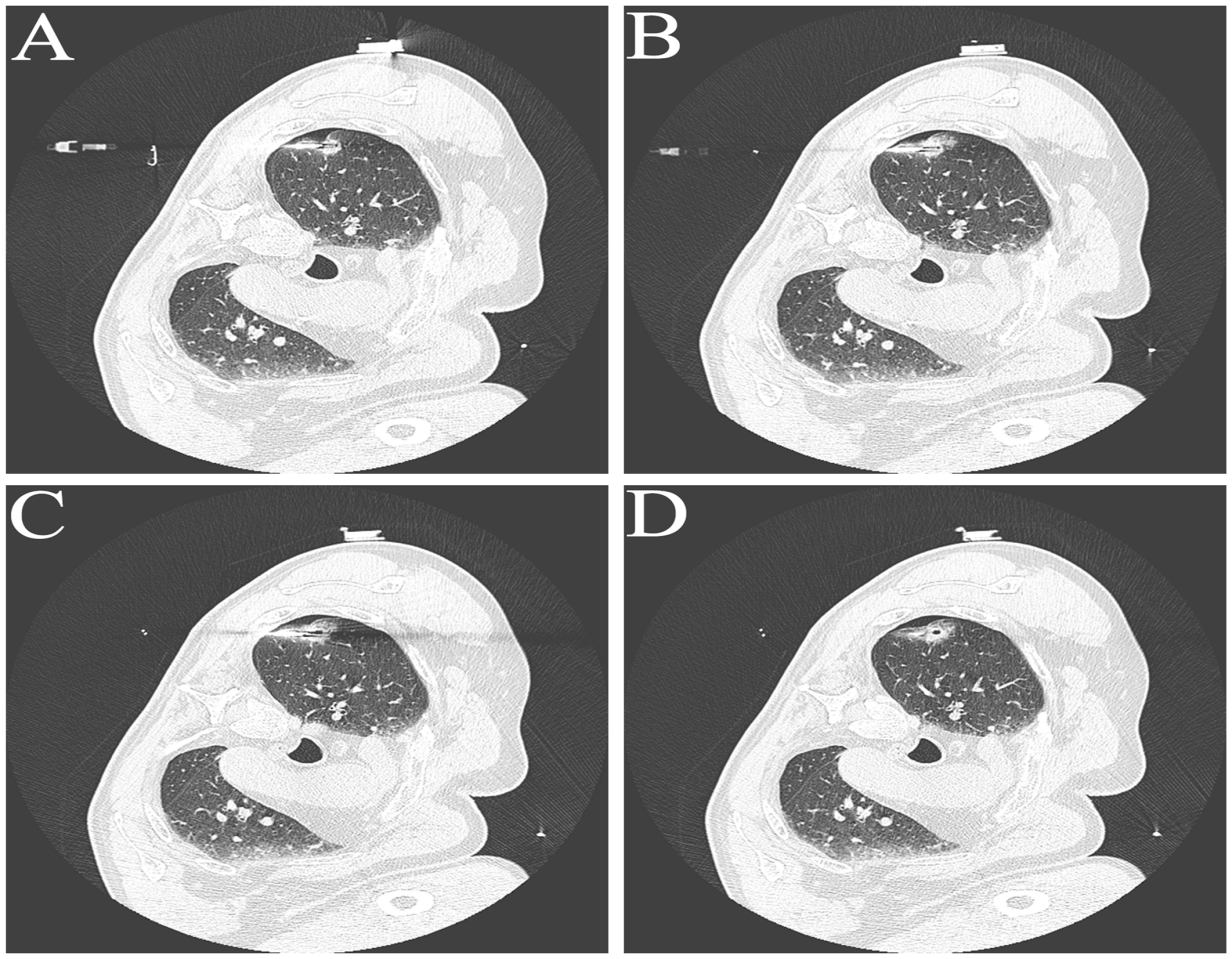
Representative Images in Laser Ablation Process. **(A-C)** Representative images of fiber optic positioning within tumor tissue during the ablation process. **(D)** Representative CT image of the tumor site after laser ablation.

**Figure 3:**
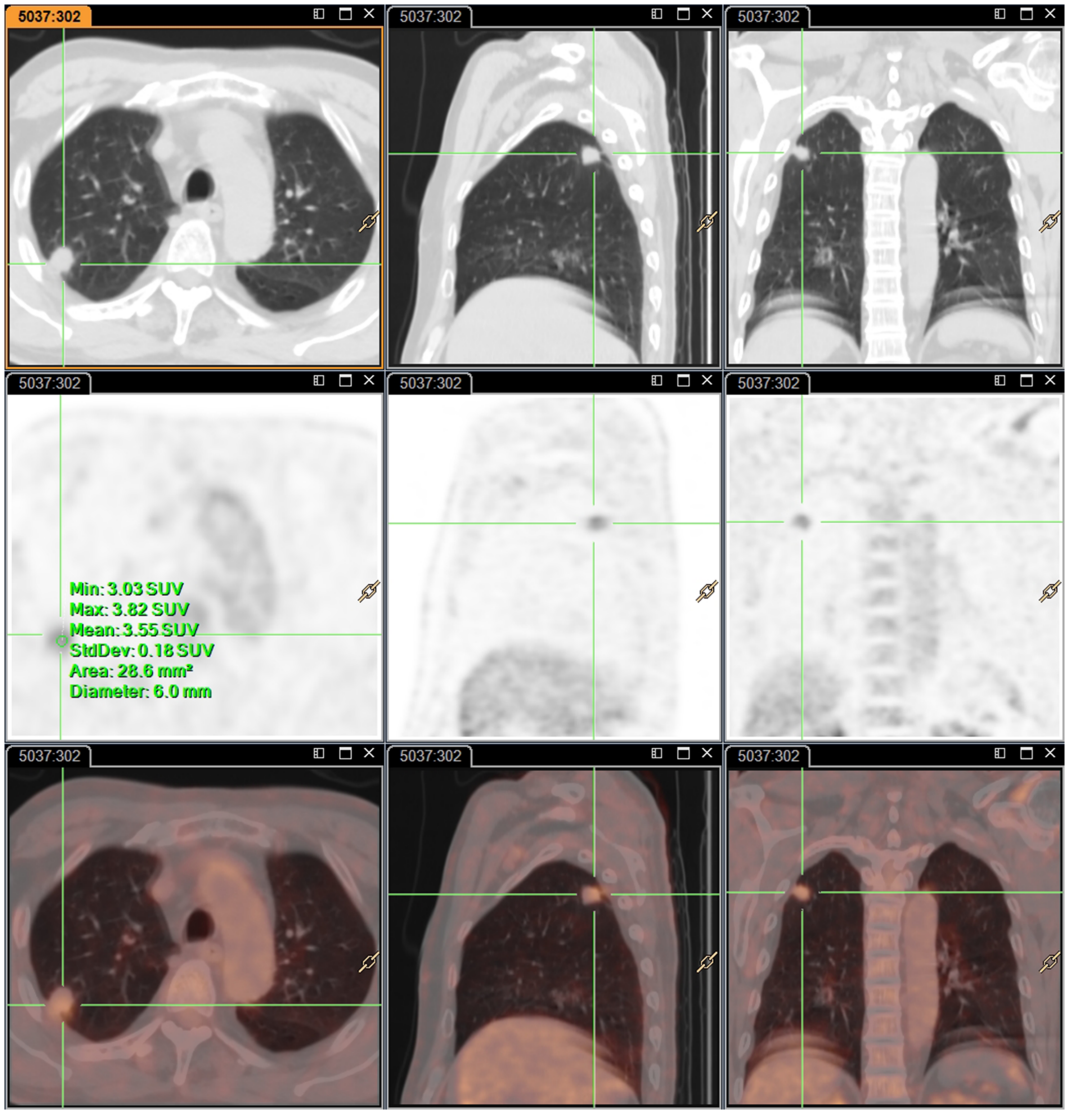
Representative images of PET-CT examination one-month post-surgery. there was no significant increase in metabolism in solid nodules in the upper lobe of the right lung.

A 63-year-old male patient, who is attending the Respiratory Department Outpatient Clinic at the Second Hospital of Hebei Medical University, was diagnosed with mixed pulmonary nodules during a physical examination in June 2019. The size of the nodules was approximately 8mm. Subsequently, he has undergone chest CT scans every six months to monitor the changes in the pulmonary nodules. Over the past four years, there has been no significant alteration in the size of the nodules. During the regular medical examination in March 2023, the size of the pulmonary nodule was measured to be 8.5mm×6.1mm (**Figure 1**). Upon re-examination in 25th September of the same year, a significant increase in the size of the pulmonary nodule was observed, measuring 19mm×18mm. Enhanced chest CT imaging suggested peripheral-type lung cancer (**Figures 1A, B**). Full communication with the patient about the condition and feasible diagnostic and therapeutic options. After personal decision by the patient, the CT-guided electromagnetic navigation system was chosen for percutaneous biopsy and laser ablation therapy. The patient has a 40-year smoking history (20 cigarettes per day) and a medical history of mild chronic obstructive pulmonary disease, with no other medical conditions and no family history of cancer. Routine preoperative blood tests, coagulation function and other examinations were performed, and the patient had no surgical contraindications. Case data are show in **Table 1**. The patient was not receiving anticoagulant medication and a preoperative intravenous infusion route was established, along with comprehensive cardiac monitoring. The patient underwent an interventional surgery on October 17, 2023. After assuming a left lateral decubitus position and exposing the site for right chest puncture, the puncture point was determined through CT scan, along with the placement of the electromagnetic navigation system locators. The skin to parietal pleura at the puncture site was infiltrated with 2% lidocaine for local anesthesia. After that, the puncture sheath was guided to the lesion site using electromagnetic navigation (**Figures 1C-E**). Remove the needle core and set the biopsy depth to 1.3cm using the biopsy device to obtain lung tissue. Rapid on-site evaluation (ROSE) during the procedure revealed atypical cells, suggestive of adenocarcinoma. Subsequently, the tissue was sent to the pathology department and the results confirmed the diagnosis of lung adenocarcinoma. Intraoperative chest CT scanning was performed to confirm the placement of the catheter at the tumor site, followed by the insertion of a 980nm wavelength semiconductor laser fiber into the appropriate position (**Figures 2A-C**). Subsequently, laser ablation therapy was performed on the lesion. Initially, a power of 7 watts (W) was applied for 30 seconds, followed by a 60-second pause, and then another 30-second application of 7 W for ablation. After ablation, a cavity can be observed at the center of the lesion on CT scan, with ground-glass opacities visible around it (**Figure 2D**). The patient tolerated the procedure well with no significant discomfort and began engaging in simple activities on the second day after surgery, which significantly reduced the burden on the body compared to surgical resection. A positron emission tomography-computed tomography (PET-CT) examination was performed 1 month after operation. The results showed that there was no significant increase in metabolism in the solid nodules in the upper lobe of the right lung, and no malignant changes were considered, suggesting complete ablation of the tumor (**Figure 3**).

**Table 1:** Case and tumor characteristics.

Sex	Age (year)	Tumor size (mm)	Pathological type	WBC (10^9/L)	RBC (10^9/L)	Platelet (10^12/L)	PT (S)	APTT (S)	INR	CEA (ng/ml)	Fiber Number	Laser wavelength	Power (W)	Energy (J)	Fellow up(month)	Examination type	Outcome
Male	63	18*19	ADC	8.89	4.38	259	12.6	32.2	1.13	6.21	1	980nm	7	420	1	PET-CT	CR

ADC, lung adenocarcinoma; WBC, white blood cell; RBC, red blood cell; PT, prothrombin time; S, second; APTT, activated partial thromboplastin time; INR, international normalized ratio; CEA, carcinoembryonic antigen; W, watt J, joule; CR, complete response.

## Discussion

Currently, thermal ablation techniques mainly include radiofrequency ablation (RFA), microwave ablation (MWA) and laser ablation (LA). During the process of RFA, tissue undergoes progressive coagulative necrosis, which is accompanied by a loss of conductivity for the radiofrequency current, resulting in an increase in impedance and limiting the ablation volume. Compared to RFA, MWA can generate higher intratumoral temperatures and a wider ablation zone, thereby reducing treatment time and improving tumor control rates. Percutaneous laser ablation has the advantages of precision, short duration, resistance to impedance effects, and real-time monitoring under MRI guidance (13). The literature on percutaneous laser ablation for the treatment of lung tumors is relatively scarce. To date, we have retrieved only six relevant reports from three medical institutions. Among these reports, only one study focuses specifically on primary lung tumors, and it was published 20 years ago. Professor Vogl from the Goethe University Hospital in Germany published the first report on percutaneous laser ablation of lung tumors in 2004. The study included a total of 30 patients, in 24 patients with pulmonary metastases, 74% of the lesions achieved complete ablation, all lesions in 6 patients with primary lung tumors achieved complete ablation (10). A retrospective comparative study demonstrated that the local tumor control rate of LA was 91% at 6 months postoperatively, slightly higher than that of RFA (85%) (14). Later, Professor Vogl conducted a comparative analysis of RFA, MWA, and LA. The three thermal ablation methods seem to be acceptable as surgical alternatives, but on the whole, MWA is the best, LA is the second, and RFA is the worst (13). This is the only comparative study of three thermal ablation methods to date; however, the reliability of the results is limited due to certain reasons: 1. This study is exclusively conducted at a single center hospital; 2. The sample size is relatively small; 3. The cases for the three treatment modalities were collected at different time periods, with cases for LA treatment collected between 2000 and 2003, while patients undergoing microwave treatment were treated between 2008 and 2014, resulting in a nearly 10-year gap between the two. During this 10-year period, there have been significant advances in operative devices, techniques, and comprehensive tumor treatment.

Dr. Weigel has also published a report on PLA for the treatment of pulmonary metastases. A total of 64 patients with pulmonary metastases underwent CT-guided percutaneous laser ablation. The median survival time of the 31 patients with complete ablation was 32.4 months. While, the median survival period of the 33 patients with partial ablation was only 12.2 months (12). The survival rate of patients with complete ablation of the lesion is significantly higher than that of patients with partial ablation of the lesion, suggesting that in future treatment, it is advisable to achieve complete ablation of the lesion as much as possible. In addition to the reports from German scholars, there is also a report from Chinese scholars. Three patients with pulmonary metastases underwent PLA under CT guidance at the First Affiliated Hospital of Zhejiang University. At follow-up examinations several months later, complete disappearance of the tumor was observed in one patient, and significant reduction of the tumor size was observed in the other two patients (15). Although the above reports have preliminarily confirmed the value of percutaneous laser ablation in the treatment of lung tumors, a larger-scale evaluation involving clinical cases is still required.

The use parameters of ablation devices directly affect the effectiveness of tumor ablation. However, there is currently no uniform standard for the use of ablation parameters in percutaneous laser ablation of lung tumors. In this study, we aimed to summarize and discuss the ablation parameters in order to provide valuable references for other researchers. Due to its high penetrability, the Nd: YAG laser with a wavelength of 1064-nanometer (nm) has always been the most widely used laser type for tumor treatment (8). A study was conducted on normal pig lungs using 1064 nm Nd: YAG laser, and it was found that ablation at 25 W for 10 minutes (15 kilojoules) resulted in the following dimensions of the ablation zone: axial diameter of 3.64 ± 0.27 cm, diametrical diameter of 2.50 ± 0.29cm, and volume of 12.07 ± 3.34cm^3^ (16). Furthermore, increasing the power beyond 25 W did not significantly increase the ablation volume. Therefore, the operator recommends selecting 25 W as the ablation setting parameter (16). Professor Vogl used a 1064 nm wavelength Nd: YAG laser for the surgery. For tumors of approximately 2 cm in size, an energy of 28 kilojoules (KJ) is applied; For tumors of approximately 3 cm in size, 45 KJ of energy is applied; For tumors of approximately 4 cm in size, 66 KJ of energy is applied; For tumors larger than 4 cm in size, an energy of 72 KJ is applied; 90% of patients tolerate the procedure well without significant side effects (10). Dr. Weigel reported that using a 14 W (laser type: Nd: YAG) to burn tumors for 15 minutes (12.6 KJ) achieved good results, but complete ablation was not achieved for metastatic tumors larger than 4 cm (11). Furthermore, the results indicate that metastases located closer to the pleura are more resistant to complete ablation compared to central metastases within the range of 1.5 to 4.0 cm (11). Professor Jiang from China achieved satisfactory results by using two optical fibers simultaneously to perform low-power ablation on transfer tumors smaller than 2 cm (5 W burning for 6 minutes, totaling 3.6 KJ, laser type: Nd: YAG) (15). Semiconductor lasers produced by laser diodes can provide wavelengths ranging from 800 nm to 980 nm, with tissue penetration similar to that of Nd: YAG laser. Due to its portability and affordability, semiconductor laser is replacing Nd: YAG laser for tumor ablation therapy and has achieved good results (9). Fielding et al. used semiconductor laser (805nm, 3W, 333s) with four x-ray guided optical fibers to perform laser ablation on pig lungs, generating a coagulative necrotic area of approximately 3.5×2.5×2 cm (17). We selected a 980nm semiconductor laser and applied a scheme of 7W laser ablation to the tumor for 30 seconds, repeated twice with a 60-second interval in between. This approach achieved complete ablation of tumors smaller than 2cm. In previous research reports, there have been certain discrepancies in the setting of ablation parameters. In the future, we will further explore the optimal ablation parameters.

High-precision control of the ablation of tumor regions without damaging surrounding healthy tissue is a challenging problem that needs to be solved in CT-guided PLA. It is also a key factor in determining the potential for wider application of this technique. However, even with the most optimal ablation parameter setting scheme, precise control remains difficult to achieve due to individual variations in tissue structure and blood flow conditions. Recently, researchers have been devoted to development of tools to assist high-temperature treatment by modeling the energy transfer of thermal ablation and the interaction between tissues to predict the temperature distribution of tissues. The specific implementation involves the following steps: 1. Generating a patient lesion structure model; 2. Calculating the power absorbed by the tissue; 3. Obtaining the tissue temperature distribution through a predictive model (9). The development of a real-time temperature monitoring tool for thermal ablation will facilitate precise control of laser ablation and provide optimal treatment outcomes for patients.

Due to the limited reports on percutaneous laser ablation for the treatment of lung tumors, we will provide a brief description of the procedural steps and some precautions. First is a brief description of the puncture process. Install patch sensors on the skin surface near the lesion, and move the patch sensors according to the puncture point aiming device prompts on the magnetic navigation interface. The red dot at the center of the aiming device on the interface of the magnetic navigation system is the target puncture point, while the green dot represents the center of the patch. Move the patch until it aligns with the red dot, which indicates the target puncture point. Once the target puncture point is located, secure the patch on the skin. During puncture, the central red dot represents the lesion point, while the green dot indicates the current puncture projection point. Adjust the puncture angle until the two points coincide and the displayed distance is close, indicating that the puncture angle has been properly adjusted. Based on the real-time needle path guidance provided by the magnetic navigation system, slowly advance the needle along the green projection direction. Preoperative preparation should include a comprehensive CT scan of the chest, electrocardiogram, echocardiogram, complete blood count, coagulation function tests, biochemical markers, etc., and discontinuation of antiplatelet and anticoagulant drugs. Additionally, emergency medications and rescue equipment (such as endotracheal intubation) should be prepared. Postoperative vital signs should be closely monitored, with attention to signs of bleeding, chest pain, and shortness of breath. A chest X-ray or CT scan should be performed within 24 hours after surgery. In addition, there may be some complications during surgery, with pneumothorax and bleeding being the most common. Most pneumothoraxes occur within 1 hour after surgery. For small pneumothoraxes, asymptomatic and stable cases, no special treatment is required. When the pneumothorax exceeds 20% or severe clinical symptoms occur, prompt closed thoracostomy should be performed for gas drainage. Mild bleeding is generally self-limiting, and patients should avoid excessive physical activity without the need for any other special treatment. When the bleeding is more severe, absolute bed rest, lateral positioning on the affected side, the use of hemostatic drugs, and, if necessary, endotracheal intubation, bronchoscopic balloon tamponade, interventional treatment, or surgical treatment may be required. Some patients may experience pleural reactions, mild cases can resolve on their own without special treatment, and operations should be stopped immediately when symptoms are severe, with adrenaline or other treatments given if necessary. In order to ensure the safety of surgeries, hospital regulatory authorities need to establish corresponding training and certification standards to ensure that doctors have sufficient skills and knowledge to operate laser devices and understand the risks and precautions of surgeries. Additionally, it is necessary to regularly assess the performance and quality of laser devices and surgical instruments.

In addition, our study has certain limitations. In this case, the tumor volume is relatively small, so the ablation efficacy for larger lesions remains uncertain. The patients we reported had no serious underlying diseases, and the safety of PLA in patients with serious underlying diseases requires further investigation.

## Conclusions

Percutaneous laser ablation under CT-ENS guidance currently has limitations such as the inability to monitor in real time, small sample size, and lack of studies on combination therapy. However, as a novel, more precise, shorter duration, impedance-independent, safe and effective minimally invasive thermal ablation method, it is expected to gain wider application and become a new alternative to surgical treatment.

## Data availability statement

The original contributions presented in the study are included in the article/supplementary material. Further inquiries can be directed to the corresponding author.

## Ethics statement

This study was approved by the Ethics Committee of the second Hospital of Hebei Medical University. The studies were conducted in accordance with the local legislation and institutional requirements. The participants provided their written informed consent to participate in this study. Written informed consent was obtained from the individual(s) for the publication of any potentially identifiable images or data included in this article.

## Author contributions

XL: Conceptualization, Data curation, Formal analysis, Investigation, Methodology, Software, Writing – original draft. SZ: Data curation, Formal analysis, Investigation, Methodology, Software, Validation, Writing – original draft. HL: Formal analysis, Investigation, Methodology, Software, Validation, Writing – original draft. XR: Data curation, Formal analysis, Investigation, Methodology, Writing – original draft. XX: Formal analysis, Software, Validation, Writing – original draft. XW: Data curation, Formal analysis, Investigation, Writing – original draft. LY: Conceptualization, Project administration, Supervision, Validation, Writing – review & editing. ZC: Conceptualization, Methodology, Project administration, Resources, Supervision, Validation, Writing – review & editing.
